# HLA-DM co-expression enhances MHC class II function in the magnetosome display system

**DOI:** 10.1128/spectrum.01653-25

**Published:** 2025-10-27

**Authors:** Ryoto Tomoe, Toru Honda, Tsuyoshi Tanaka, Tomoko Yoshino

**Affiliations:** 1 Division of Biotechnology and Life Science, Institute of Engineering, Tokyo University of Agriculture and Technology 13125, Koganei, Tokyo, Japan; Emory University School of Medicine Biochemistry, Atlanta, Georgia, USA

**Keywords:** MHC class II, HLA-DM, magnetosome display system

## Abstract

Efficient identification of antigenic peptides that bind to major histocompatibility complex class II (MHC II) molecules is crucial for vaccine and therapeutic development. However, mammalian expression systems are constrained by high costs and low scalability, particularly for stable peptide exchange. To address these challenges, we developed a bacterial magnetosome surface display system using *Magnetospirillum magneticum*, a magnetotactic bacterium that produces lipid bilayer-coated magnetosomes. This system enables the co-expression of MHC II and human leukocyte antigen DM (HLA-DM) on magnetosome surfaces via cohesin–dockerin interactions. This spatial co-localization significantly enhances MHC II stabilization and immobilization on magnetosomes. Moreover, co-expression with HLA-DM improves functional MHC II peptide loading, as demonstrated by increased binding of the influenza-derived HA₃₀₆–₃₁₈ epitope. Structural analysis suggests that HLA-DM stabilizes MHC II by facilitating peptide loading and reducing degradation. Overall, this system provides a robust alternative to mammalian expression systems and represents a promising tool for high-throughput antigen discovery and rational vaccine design.

**IMPORTANCE:**

High-throughput screening of MHC II–peptide interactions remains a major bottleneck in antigen discovery. Conventional mammalian systems are costly and time-consuming, which limits scalability. This study introduces a bacterial system that enables stable co-localization of MHC II and human leukocyte antigen DM (HLA-DM) on genetically engineered magnetosomes. The system supports functional pMHC II complex formation and epitope screening by promoting peptide exchange and conformational stability. It integrates expression, immobilization, and peptide loading in a single step, and it is compatible with diverse immunological targets, offering broad applications in vaccine development and immunotherapy.

## INTRODUCTION

Major histocompatibility complex class II (MHC II) molecules are membrane proteins expressed on specific antigen-presenting cells, such as B cells, dendritic cells, and macrophages (1). These molecules present pathogen-derived antigenic peptides on the cell surface as peptide/MHC II (pMHC II) complexes to initiate adaptive immunity. The pMHC II complexes activate CD4^+^ T cells via the T-cell receptor, triggering cytokine secretion and various immune responses (2–4). MHC II molecules are implicated in the pathogenesis of several diseases, including cancer (5), infectious diseases (6), and autoimmune disorders (7, 8). Therefore, identifying antigenic peptides that bind to MHC II molecules is essential for therapeutic and vaccine development (9).

Peptide mapping is a common strategy to identify antigenic peptides that bind MHC II molecules. It enables systematic screening by generating overlapping peptide sequences that span entire open reading frames (ORFs) of viral or host proteins (10–12). These peptides are tested for MHC binding by forming pMHC complexes and evaluating their stability via fluorescence-activated cell sorting (FACS). Although this approach enables comprehensive analysis of potential antigenic regions, it is time-consuming and labor-intensive. Consequently, high-throughput screening of 100–2,000 candidate peptides with MHC II molecules is often required for antigen discovery. This process requires highly efficient pMHC II complex formation, which in turn depends on improving both the productivity and functional stability of MHC II molecules.

Enhancing MHC II functionality depends on efficient peptide exchange within the peptide-binding groove. MHC II molecules are initially loaded with class II-associated invariant chain peptide (CLIP), a low-affinity peptide that must be replaced with higher-affinity antigenic peptides for effective immune recognition (13). The chaperone-like protein, DM (human leukocyte antigen DM) facilitates this process by dissociating CLIP and promoting exchange for higher-affinity antigenic peptides through co-localization with MHC II molecules via their transmembrane regions (14, 15). In the absence of DM, empty MHC II molecules aggregate and lose functionality due to their instability and inability to bind antigenic peptides (16, 17). Therefore, close spatial association between MHC II and DM is essential for efficient antigen loading and stable pMHC II complex formation.

To address this, we developed a novel strategy to co-localize MHC II and DM on genetically engineered magnetosomes derived from the magnetotactic bacterium *Magnetospirillum magneticum*. This prokaryotic microorganism produces magnetosomes, magnetite (Fe_3_O_4_) nanocrystals enclosed in a lipid bilayer membrane enriched with specific proteins (18). Genetically functionalized magnetosomes have been applied in various biotechnological fields, including immunoassays, biosensors, drug delivery, enzyme reactions, and ligand-receptor interaction analyses (19–22). We previously established the “magnetosome display system,” a technology that enables the surface expression of target proteins on magnetosomes (23). We have recently expanded this system to enable controlled localization of multiple proteins within nanoscale proximity (24). By leveraging this approach, we aim to facilitate the functional expression of MHC II molecules via spatial co-localization with DM on magnetosomes. This system provides a low-cost, scalable method to produce stable pMHC II complexes and holds promise for high-throughput antigenic peptide screening.

## MATERIALS AND METHODS

### Bacterial strains and growth conditions

Gene cloning experiments were performed using *Escherichia coli* TOP10 (Life Technologies, CA, USA). *E. coli* transformants were grown at 37°C on Luria-Bertani agar plates or in liquid containing 50 µg/mL ampicillin for 16-18 h. *M. magneticum* AMB-1 (ATCC 700264) *lon* gene deletion mutant (AMB-1 Δ*lon*) was used for recombinant protein expression. AMB-1 Δ*lon* was cultured, as described previously (25). Briefly, AMB-1 Δ*lon* was cultured in magnetic spirillum growth medium (MSGM) (26) supplemented with gentamycin (2.5 µg/mL) at 28°C under microaerobic conditions. AMB-1 Δ*lon* transformants were cultured in MSGM containing gentamycin (2.5 µg/mL) and ampicillin (5 µg/mL). During the mid-log phase, recombinant protein expression was induced with 500 ng/mL anhydrotetracycline hydrochloride (ATc, Kanto Chemical, Tokyo, Japan).

### Plasmid construction

A schematic representation of the co-expression plasmids constructed in this study is presented in Fig. S1. The co-expression plasmids consist of two fusion gene sets: *mms13-cohesin* and *target-dockerin* genes. The *mms13-cohesin* gene encodes Mms13, an (N_4_S)_18_ linker, and cohesin domains from *Clostridium thermocellum* (CohC, amino acids 557-702 of CipA) and *Ruminococcus flavefaciens* (CohR, amino acids 29-186 of ScaB), expressed under the control of the P_mms16_ promoter. The *target-dockerin* genes encode the dockerin domains from *C. thermocellum* (DocC, amino acids 673-741 of CelS) and *R. flavefaciens* (DocR, amino acids 793-879 of ScaA). These dockerin domains were fused to CLIP (PVSKMRMATPLLMQA)-conjugated MHC II (pMHC II-DocC) and HLA-DM (DM-DocR), under the control of P_msp1(tetO)_. The human MHC II allele HLA-DRB1 was used in this study.

The tetracycline-inducible expression vector pUMtOR (27) was modified by introducing a fusion gene of CohC and CohR into the subsp. site, yielding pUMtOR-CohCR (Table S1). To enable pMHC II and DM co-expression, the pMHC II-DocC fusion gene was inserted into the *HpaI* site of pUMtOR-CohCR, generating pUMtOR-CohCR-pMHC II. The DM-DocR fusion gene was subsequently introduced into the *BstZ17I* site of pUMtOR-CohCR-pMHC II to construct pUMtOR-CohCR-pMHC II/DM. The pMHC II-DocC construct consists of CLIP-(G_4_S) linker-Factor Xa cleavage site-(G_4_S) linker-MHC II β chain (30-221 amino acids)-(G_4_S)_3_ linker- MHC II α chain (amino acids 26-206)-DocC-FLAG tag. The DM-DocR construct includes the DM β chain (amino acids 19-218)-(G_4_S)_4_ linker-DM α chain (amino acids 27-233)-DocR-His tag. Additionally, cysteine 46 in the DM β chain was replaced with serine. pMHC II-DocC and DM-DocR genes were organized into an operon using a ribosome-binding site with the sequence aaagagaggagataccaat. Plasmid vectors were introduced into AMB-1 Δ*lon* via electroporation, as described previously (25).

### Preparation of magnetosomes

After ATc addition, AMB-1 Δ*lon* transformants were collected via centrifugation (9000 × *g* for 10 min at 4°C). Cell pellets were resuspended in HEPES-Ca buffer (10 mM, 1 mM CaCl_2_, pH 7.4), and disrupted using a French press at 1500 kg/cm^2^ (Ohtake Works, Tokyo, Japan). Magnetosomes were isolated from disrupted cells using a columnar neodymium-boron (Nd-B) magnet. The magnetosomes were magnetically separated and washed with HEPES-Ca buffer for 10 cycles. Magnetosome concentration in suspension was determined via optical density (OD) at 660 nm using a UV-2200 spectrophotometer (Shimadzu, Kyoto, Japan). An OD of 1.0 corresponded to 172 µg magnetosomes (dry weight) per mL. Magnetosomes derived from transformants harboring pUMtOR-CohCR-pMHC II and pUMtOR-CohCR-pMHC II/DM were referred to as pMHC II-mag and pMHC II/DM-mag, respectively.

### Enzyme-linked immunosorbent assay (ELISA)

Magnetosomes (50 µg) were incubated with 1 µg/mL ALP-conjugated anti-FLAG IgG antibody (Sigma-Aldrich, MO, USA) in TBS-T (Tris-buffered saline containing 0.1% Tween-20, pH 7.4) for 30 min at room temperature (20°C–27°C) to detect immobilized MHC II-DocC. Subsequently, magnetosomes were magnetically separated, the supernatant was removed, and the collected magnetosomes were washed three times with 200 µL of TBST under sonication using a bath-type sonicator (Honda Electronics Co., Ltd, Aichi, Japan). This washing step was sufficient to prevent non-specific signals. After washing, magnetosomes were resuspended in TBS, and 50 µL of Lumi-Phos 530 (Wako Pure Chemical Industries, Tokyo, Japan) was added as the luminescence substrate. The mixture was incubated for 5 min at room temperature, and luminescence intensity was measured using an SH-9000 microplate reader (Corona Electric, Ibaraki, Japan). The amount of DM-DocR immobilized on the magnetosomes was determined using 1 µg/mL Rabbit anti-His tag IgG (Abcam, MA, USA) and 1 µg/mL goat ALP-conjugated anti-rabbit IgG (Thermo Fisher Scientific Inc., MA, USA), following the same procedure described above. All ELISA measurements were performed in triplicate. Data are presented as mean ± SD, and the statistical tests applied to each figure are specified in the corresponding legends.

### Evaluation of CLIP/MHC II complex formation

Magnetosomes (50 µg) were incubated with 100 µL of peptide binding buffer (citrate-phosphate buffer, 2 mM CaCl_2_, pH 5.5) containing 1.25, 2.5, 5, and 10 µg/mL Factor Xa for 90 min at 37°C to digest the Factor Xa site between CLIP and MHC II. Subsequently, the magnetosomes were washed three times with 200 µL TBST. Thereafter, 100 µL of 10 µg/mL mouse L243 IgG (Meridian Life Science Inc., ME, USA), which specifically recognizes properly folded MHC II αβ heterodimers, was added and incubated for 1 h at room temperature (28). After washing with TBST, 100 µL of 1 µg/mL ALP-labeled anti-mouse IgG antibody was added and incubated for 30 min at room temperature. Subsequently, the magnetosomes were resuspended in 50 µL of TBS, followed by the addition of 50 µL of Lumi-phos 530. Luminescence intensity was measured after a 5 min reaction at room temperature.

### Antigen peptide binding assay

Magnetosomes (50 µg) were incubated with 50 µM HA_306-318_(PKYVKQNTLKLAT) conjugated to biotin in peptide binding buffer for 90 min at 37°C. After washing with TBS-T, 100 µL of 1 µg/mL ALP-conjugated streptavidin (Life technologies, MA, US) was added to the magnetosome mixture. The magnetosomes were then resuspended in 50 µL TBS, followed by the addition of 50 µL of Lumi-Phos 530. After 5 min of incubation, luminescence was measured using a microplate reader. Specific binding was calculated by subtracting the signal from wild-type magnetosomes from that of transformant magnetosomes, and then dividing by the ALP-anti-FLAG tag IgG signal intensity.

## RESULTS

### Immobilization of MHC II and HLA-DM onto magnetosomes using the cohesin-dockerin interaction

**Fig 1:**
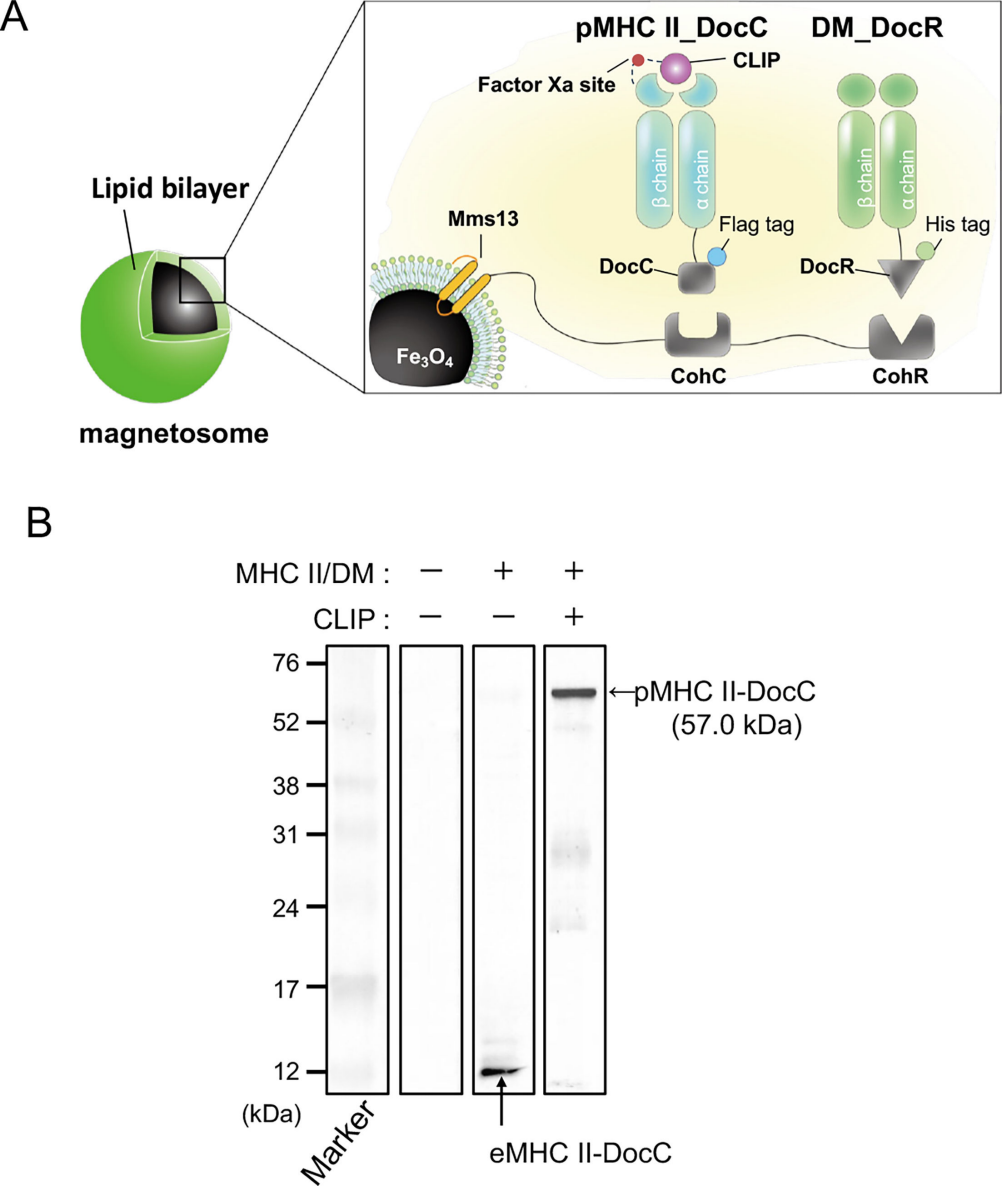
Functional immobilization of MHC II onto magnetosomes.(**A**) Schematic overview of MHC II and HLA-DM immobilization on magnetosomes using the cohesin–dockerin interactions. (**B**) Western blot analysis using an anti-FLAG tag antibody targeting the C-terminus of MHC II-DocC on the magnetosome membrane derived from transformants induced with eMHC II + DM or pMHC II + DM. Expected molecular weights: pMHC II-DocC, 57.0 kDa; eMHC II-DocC, 54.6 kDa.

The spatial proximity of DM is crucial for promoting peptide exchange on MHC II molecules. In this study, MHC II and DM were co-localized on magnetosomes via cohesin–dockerin interactions using the magnetosome display system (24, 29). Fig. 1A illustrates the genetic design of the transformants. In this configuration, MHC II was fused to the dockerin domain DocC and DM to DocR. The corresponding cohesin domains, CohC and CohR, were fused to Mms13, a magnetosome-targeting protein.

Western blot analysis revealed distinct differences depending on the peptide-loading status of MHC II. A 12-kDa band was observed in the eMHC II/DM-mag sample, likely representing a degradation fragment of unbound MHC II. In contrast, the pMHC II/DM-mag sample showed a 60-kDa band consistent with the predicted size of full-length, peptide-bound MHC II (Fig. 1B). No specific bands were detected in the wild-type control. Unbound eMHC II, lacking the stabilizing CLIP peptide, is unstable and prone to aggregation and degradation (30). The predicted molecular weight of eMHC II-DocC was 54.6 kDa, consistent with the appearance of a 12-kDa degradation product. Meanwhile, the 60kDa band observed in the pMHC II/DM-mag sample closely matched the expected size of pMHC II-DocC (57.0 kDa), supporting its stability upon peptide loading. These results suggest that stable MHC II expression on magnetosomes requires peptide binding.

### Proximity to HLA-DM promotes proper folding and immobilization of MHC II on magnetosomes

**Fig 2:**
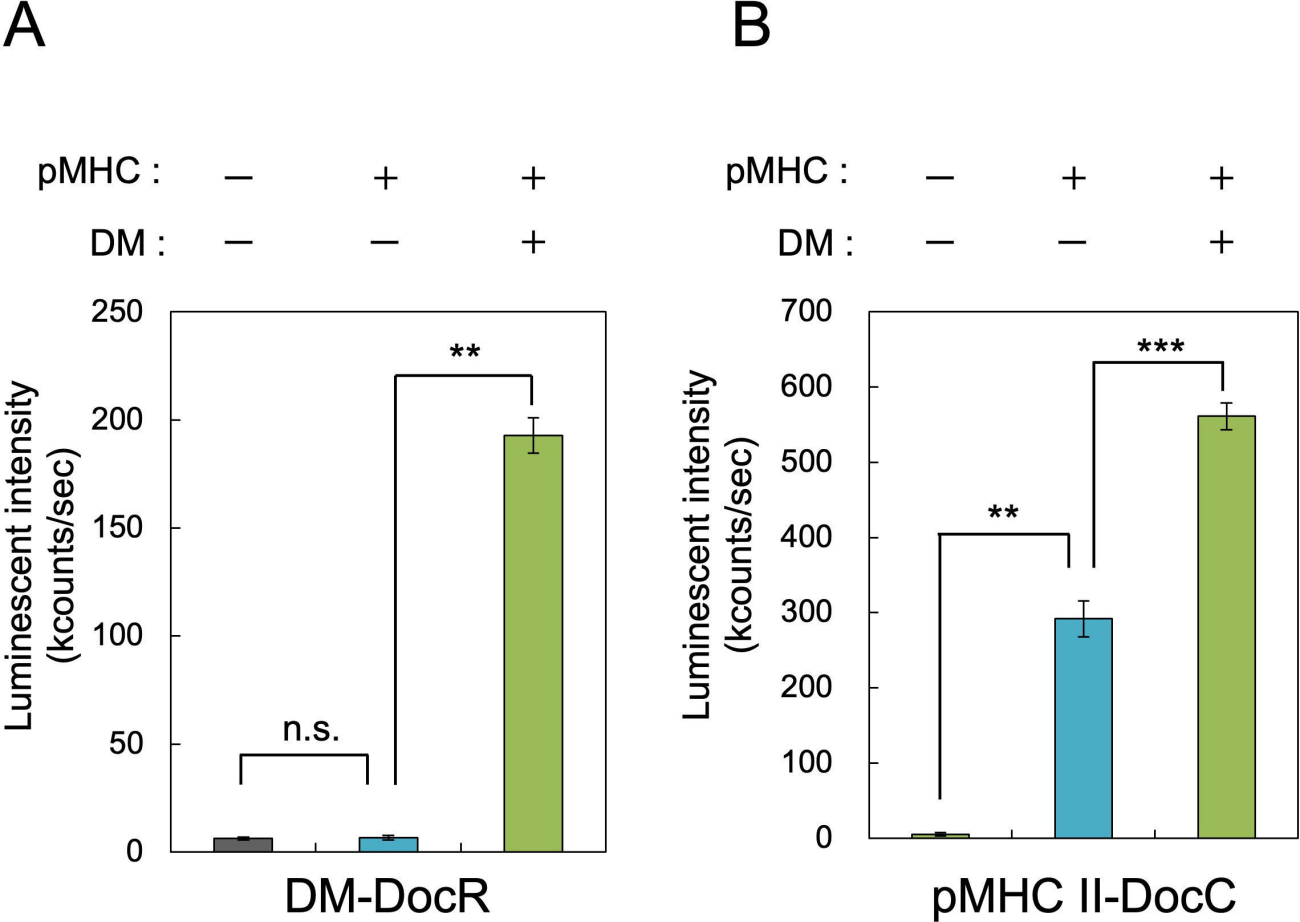
ELISA analysis of pMHC II-DocC and DM-DocR on magnetosomes derived from WT and transformants induced with pMHC II or pMHC II + DM. (**A**) Fluorescence-based evaluation using an anti-His tag antibody targeting the C-terminus of DM-DocR. (**B**) Fluorescence-based evaluation using an anti-FLAG tag antibody targeting the C-terminus of pMHC II-DocC. Error bars represent the standard deviation calculated from three independent experiments. Data are shown as mean ± SD (*n* = 3 independent biological replicates). Statistical significance was evaluated using a two-tailed Welch’s *t*-test; *P < 0.05, **P < 0.01, ***P < 0.001, ns, not significant.

The role of DM in promoting immobilization and proper folding of MHC II was evaluated by quantifying pMHC II-DocC on magnetosomes, with or without DM co-expression, using antibody-based assays. Fixation of the fusion protein DM-DocR to magnetosomes was first confirmed by detecting the C-terminus His tag. Strong luminescence in the pMHC II/DM-mag sample, compared with controls (Fig. 2A), confirmed efficient recruitment of DM-DocR via CohR interaction.

Next, the immobilization level of pMHC II–DocC, fused with a FLAG tag at the C-terminus, was assessed. Luminescence signals were detected in both the pMHC II-mag and pMHC II/DM-mag samples. Notably, the signal from pMHC II/DM-mag was approximately twice that of pMHC II-mag (Fig. 2B). This result indicated that co-expression with DM enhances the surface display of pMHC II on magnetosomes, likely by promoting proper folding and stabilization via DM proximity.

**Fig 3:**
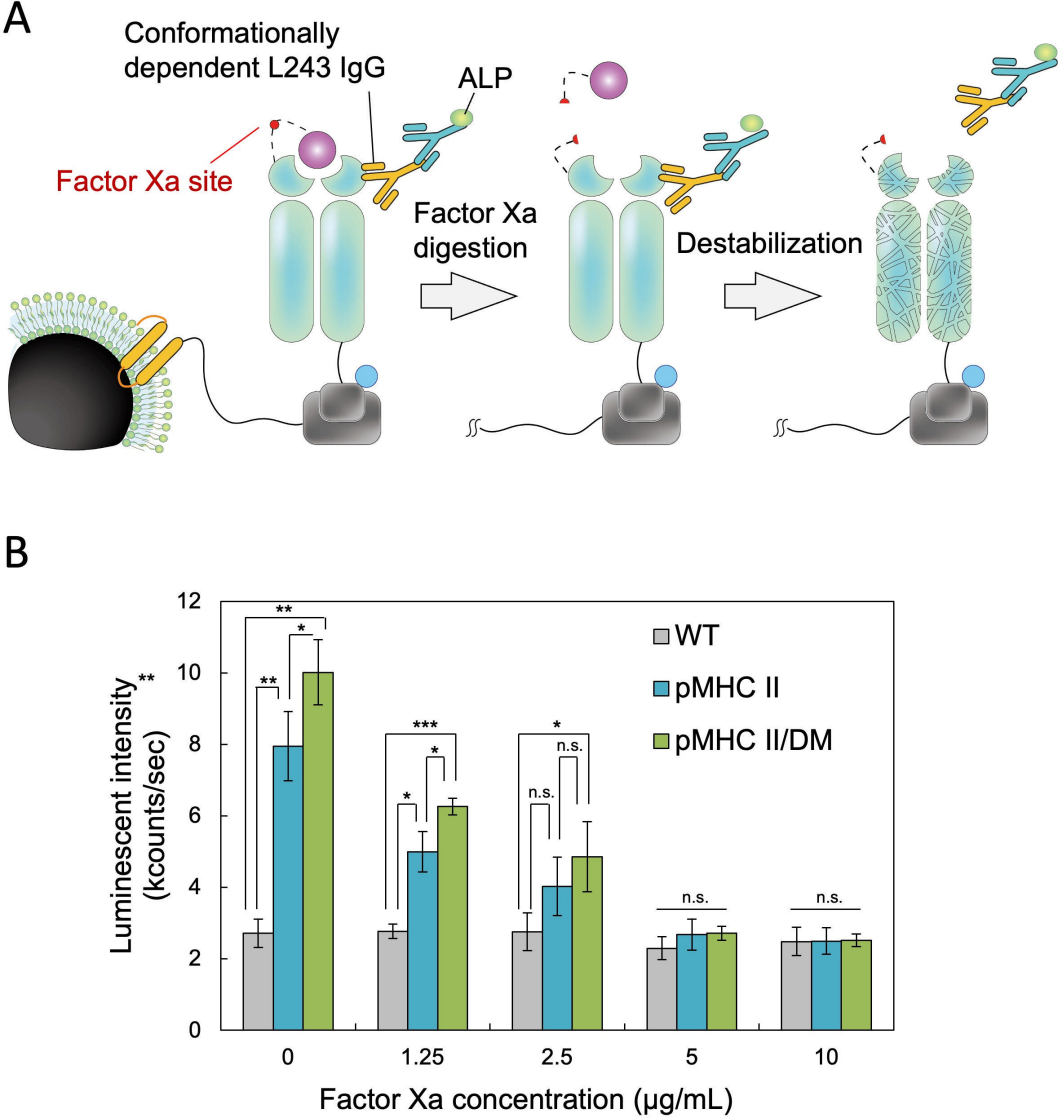
MHC II evaluation on magnetosomes forming a complex with CLIP using the DM specific antibody. (**A**) Schematic illustration of pMHC II-DocC reorganization on the magnetosomes using the protein conformation-dependent L243 antibody. (**B**) Antigen binding assay with L243 IgG. WT magnetosomes and transformants harboring pMHC II or pMHC II/DM were pretreated with various concentrations of Factor Xa. The binding signal of the L243 antibody was detected using an ALP-conjugated secondary antibody. Data are shown as mean ± SD (*n* = 3 independent biological replicates). Statistical significance was evaluated using a two-tailed Welch’s *t*-test; *P < 0.05, **P < 0.01, ***P < 0.001, ns, not significant.

To assess complex formation between magnetosome-displayed MHC II and CLIP, we used the conformation-specific antibody L243 IgG (Fig. 3A), which recognizes properly folded MHC II αβ heterodimers (28). ELISA results revealed significantly higher luminescence in the pMHC II/DM-mag sample than in pMHC II-mag (Fig. 3B; 0 µg/mL) (*P* < 0.05), indicating improved complex formation upon DM co-expression. Next, Factor Xa was used to cleave the linker between CLIP and MHC II to assess whether CLIP removal destabilizes MHC II. Increasing Factor Xa concentrations caused a dose-dependent decrease in luminescence, reaching wild-type levels at >5 µg/mL. This reduction suggested structural destabilization of MHC II following CLIP removal. Collectively, these findings demonstrate that MHC II expressed on magnetosomes forms a stable complex with CLIP and retains its native conformation. DM co-expression enhances both the immobilization efficiency and structural integrity of pMHC II on magnetosome surfaces.

### HLA-DM enhances peptide binding to MHC II on magnetosomes

MHC II peptide-binding capability expressed on magnetosomes was assessed using a biotinylated helper epitope HA_306–318_ (PKYVKQNTLKLAT), derived from influenza hemagglutinin (31). HA is the primary target of neutralizing antibodies (32) and the most abundant surface protein on influenza virions (33). Enhancing HA-MHC II interactions may aid in identifying conformational epitopes involved in antibody generation, thereby contributing to influenza vaccine development. Therefore, HA_306–318_ was selected as a model peptide to evaluate whether DM facilitates CLIP exchange on magnetosome-displayed MHC II.

**Fig 4:**
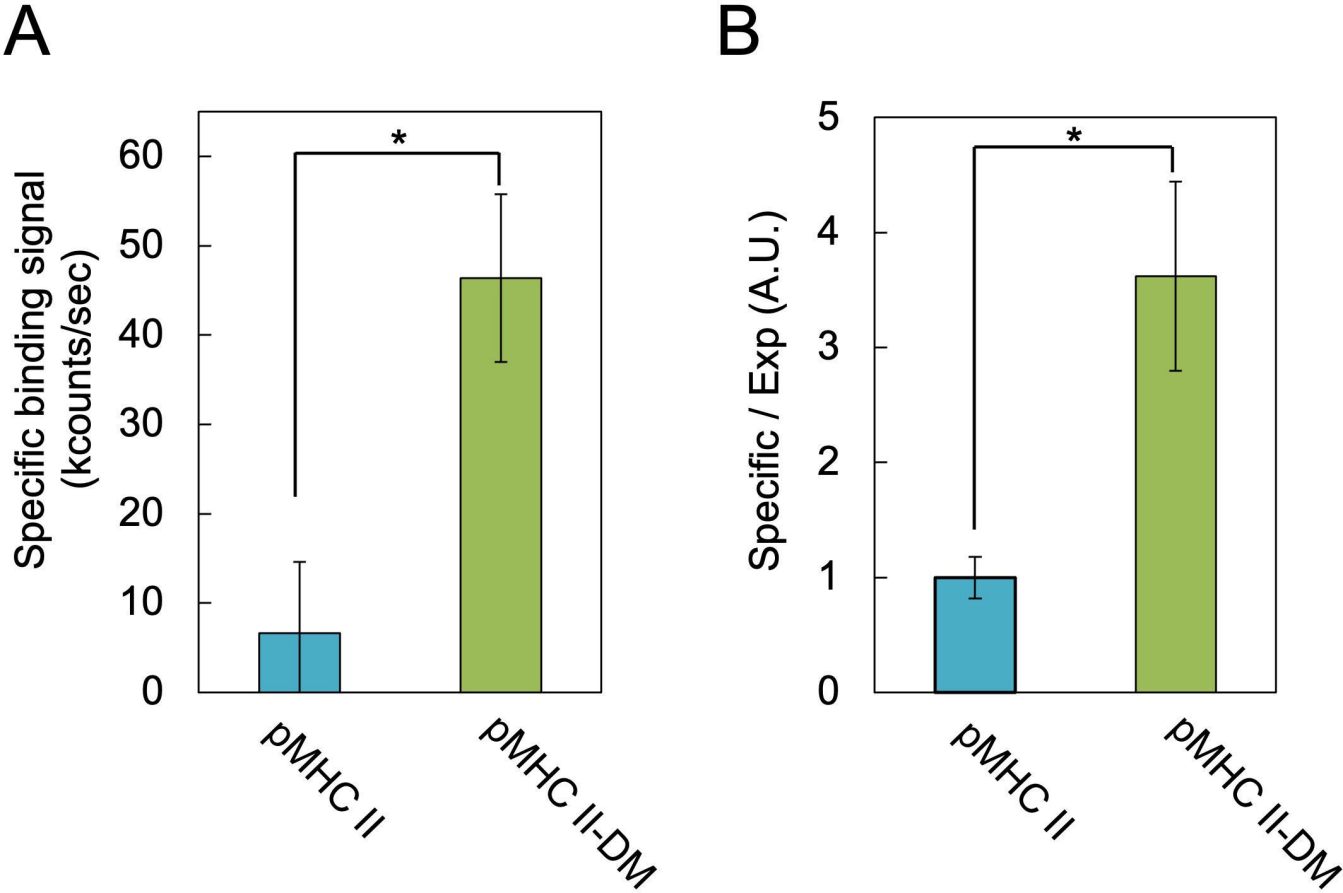
Antigen peptide binding assay using biotinylated HA_306–318_. (**A**) Fluorescence was evaluated using ALP-conjugated streptavidin. The specific binding signal was calculated by subtracting non-specific binding signal of WT-mag from that of each recombinant magnetosome. (**B**) The specific/Exp ratio was calculated by dividing the specific binding signal value of HA by the immobilization signal of pMHC II-DocC (Fig. 3B). Values for pMHC II/DM bound to the substrate were normalized relative to the value for pMHC II. Data are shown as mean ± SD (*n* = 3 independent biological replicates). Statistical significance was evaluated using a two-tailed Welch’s *t*-test; *P < 0.05.

Luminescence measurements revealed significantly higher signals for pMHC II/DM-mag than pMHC II-mag (Fig. 4A). After normalization to pMHC II immobilization levels (Fig. 3), the peptide-binding capability of pMHC II/DM-mag was approximately threefold greater than that of pMHC II-mag (Fig. 4B). These results indicate that co-expression of DM-DocR promotes CLIP exchange with the biotinylated HA_306–318_ peptide, thereby enhancing the functional peptide-loading efficiency of MHC II on magnetosomes.

## DISCUSSION

Antigen presentation by MHC class II requires efficient peptide exchange, facilitated by the non-classical chaperone HLA-DM. Reconstructing this coordinated mechanism in a stable and isolatable system has been challenging. In this study, we engineered magnetosomes derived from magnetotactic bacteria to co-display peptide-loaded MHC II (pMHC II) and HLA-DM using orthogonal cohesin–dockerin interactions. This configuration (i) doubled the amount of correctly folded pMHC II on the magnetosome surface, (ii) suppressed degradation of empty MHC II, and (iii) enhanced peptide exchange, resulting in a threefold increase in HA_306–318_ epitope binding compared with MHC II alone. These findings establish magnetosomes as a modular, genetically programmable platform for assembling chaperone-assisted pMHC II complexes.

Identifying antigenic peptides that bind to MHC II is critical for vaccine and therapeutic development, yet high-throughput peptide screening requires both stable MHC II production and functional peptide loading. In mammalian systems, dendritic cell activation significantly extends MHC II half-life, with activated cells expressing up to 2 million MHC II molecules on their surface (34). However, large-scale MHC II production in mammalian cells is limited by slow growth and high cost. In contrast, bacterial hosts offer rapid growth and efficient protein production, but functional MHC II expression remains rare owing to the lack of an appropriate display system.

Recombinant MHC II molecules are inherently unstable in the absence of bound antigen peptides (35). *In vivo*, MHC II is stabilized by CLIP, a low-affinity peptide that dissociates rapidly under physiological conditions (36). Co-expression of DM, which facilitates peptide exchange, helps maintain MHC II stability. We used the magnetosome display system to co-localize DM and MHC II via cohesin–dockerin interactions to enable this function in bacteria. This co-localization strategy increased the amount of MHC II immobilized on magnetosomes by approximately twofold (Fig. 2B). The increase likely results from DM-mediated promotion of CLIP re-binding following dissociation from unstable pMHC II complexes (37).

Structural instability is a hallmark of unoccupied MHC II molecules, particularly in the α-subunit 3₁₀ helix, β2 Ig-like domain, and β-subunit helical region (residues 62–71) (38). Peptide dissociation renders these regions susceptible to unfolding and degradation. CLIP cleavage by factor Xa significantly reduced detection by a conformation-specific antibody (Fig. 3B), indicating that peptide dissociation disrupts MHC II structure and triggers degradation. These results highlight the need for DM co-expression to stabilize peptide loading and prevent conformational collapse. In the presence of DM, HA binding per magnetosome increased by approximately threefold. DM promotes peptide exchange by selectively binding to MHC II, inducing structural modifications that expose the peptide-binding groove and facilitate peptide replacement. While HA can bind MHC II in the absence of DM to a limited extent, co-expression of DM markedly enhanced peptide loading, highlighting its functional importance. Although HA_306–318_ served as a model epitope in this study, DM-mediated peptide exchange is peptide-specific, underscoring the value of magnetosome-displayed MHC II for high-throughput screening of antigenic peptides sensitive to DM-mediated exchange. Negative peptide controls were not included in this study, as our primary focus was on HLA-DM–mediated enhancement; future studies will incorporate such controls to more rigorously evaluate peptide specificity.

Co-localizing MHC II and DM in a modular manner enables systematic evaluation of diverse peptide libraries in a scalable, cost-efficient bacterial host. Although the present work focuses on influenza epitopes, the same approach could be extended to tumor-associated and autoimmune epitopes, in which peptide exchange efficiently shapes immunogenicity. Additionally, the magnetosome display platform is compatible with other membrane protein systems, including MHC class I molecules, T-cell receptors (TCRs), and even G-protein-coupled receptors (GPCRs), enabling broader applications in immunology and membrane biology. In fact, the magnetosome display concept has been adapted to other protein families: we previously displayed single-chain MHC I/peptide complexes recognized by cognate TCRs (39), tropomyosin receptor kinase (40), and GPCRs (23). At the same time, target-wise optimization remains essential, particularly given differences between bacterial and eukaryotic systems (e.g., glycosylation state and membrane composition). The magnetosome membrane differs from the lipid composition of mammalian endosomal membranes, which may influence peptide selection and exchange efficiency. Notably, we previously reported that modifying the magnetosome membrane to more closely mimic human lipid environments significantly improved ligand binding to a model GPCR (41), suggesting that a similar strategy may enhance MHC II functionality. Furthermore, glycosylation of the MHC II α- and β-chains may be necessary for full biological activity. Although prokaryotic glycoengineering has been reported for eukaryotic proteins (42), integrating such modifications into the magnetosome system will likely be essential for achieving full receptor functionality. Addressing these limitations through expanded epitope validation, advanced membrane remodeling, and post-translational engineering will be key to realizing the system’s full potential for immunological applications.

Advances in mass spectrometry and machine learning are improving MHC-peptide binding predictions (43). Combining such tools with data from this platform may accelerate epitope discovery and vaccine development (44). Conventional MHC II tetramers—produced by soluble refolding of α/β chains with a predefined peptide, followed by biotin–streptavidin multimerization—remain the gold standard for detecting antigen-specific CD4^+^ T cells by flow cytometry, but they require prior peptide selection and offer little flexibility for post-assembly exchange (45). Other workflows (e.g., yeast or mammalian display, recombinant refolding) share similar constraints regarding scalability and membrane context. In contrast, our magnetosome-based system provides genetic encoding, magnetic enrichment, and optional HLA-DM co-localization. Although we did not perform a library-scale screen here, the format is amenable to high-throughput, plate-based magnetic handling coupled with LC–MS/MS or sequencing readouts, which we plan to implement in future work.

In conclusion, this study presents a scalable, genetically encoded system for the co-expression of MHC II and HLA-DM on bacterial magnetosomes. The platform supports efficient peptide exchange and MHC II stabilization, providing a foundation for high-throughput antigen screening and potential applications in immunotherapy and vaccine design.

## SUPPLEMENTAL MATERIAL

Supplemental figures and table spectrum.01653-25-s0001.docx Figures S1 and S2, and Table S1.

ASM does not own the copyrights to Supplemental Material that may be linked to, or accessed through, an article. The authors have granted ASM a non-exclusive, world-wide license to publish the Supplemental Material files. Please contact the corresponding author directly for reuse.
