# Supplementary material for: HLA-DM co-expression enhances MHC class II function in the magnetosome display system
Source: Microbiol Spectr. 2025 Oct 27;13(12):e01653-25. doi: 10.1128/spectrum.01653-25 (PMC12671180; doi:10.1128/spectrum.01653-25)
Supplement: Supplemental figures and table — Figures S1 and S2, and Table S1. [file spectrum.01653-25-s0001.docx]

HLA-DM Co-Expression Enhances MHC Class II Function in the Magnetosome Display System

Ryoto Tomoe, Toru Honda, Tsuyoshi Tanaka, and Tomoko Yoshino#

Division of Biotechnology and Life Science, Institute of Engineering, Tokyo University of Agriculture and Technology, Koganei, Tokyo, Japan

Running Head: Magnetosome Display Platform for Vaccine Development

#Address correspondence to Tomoko Yoshino, [y-tomoko@cc.tuat.ac.jp](mailto:y-tomoko@cc.tuat.ac.jp)


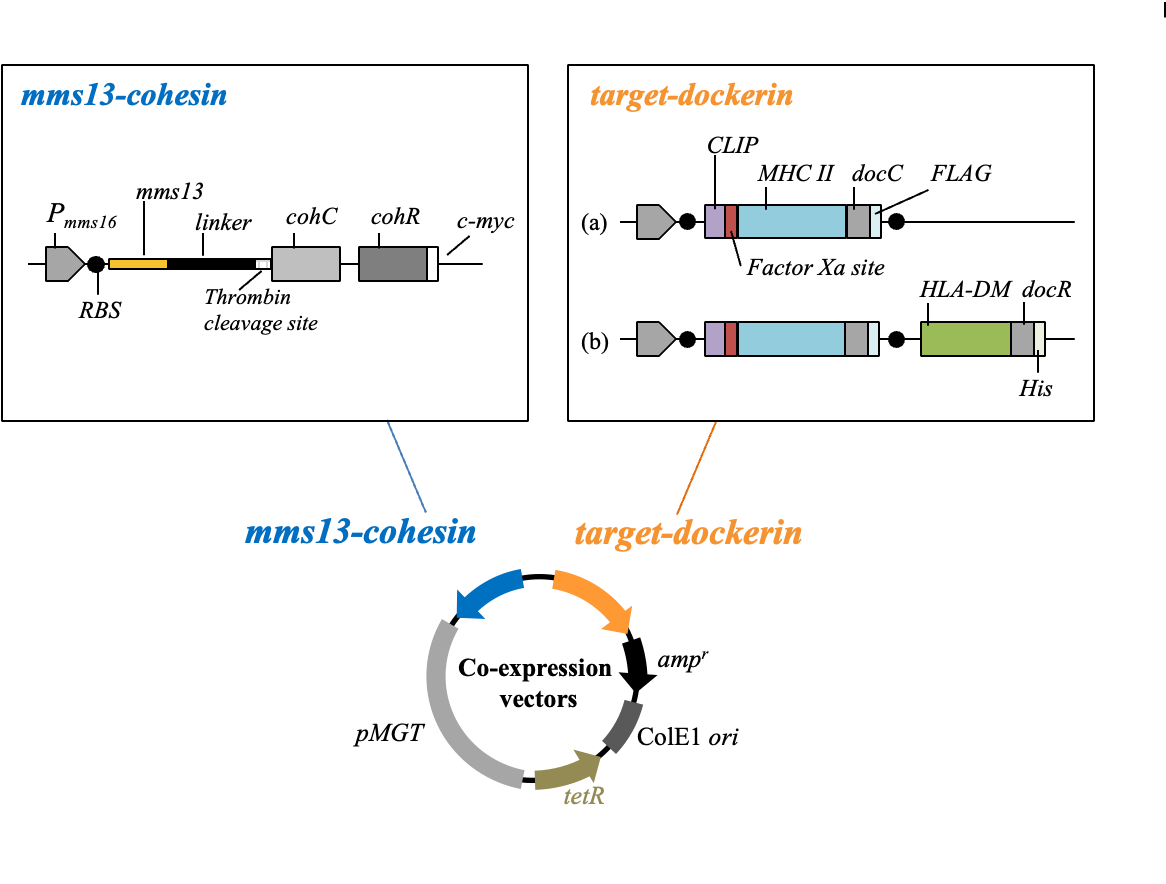


Fig. S1 Vector map of co-expression plasmids pUMtOR-CohCR-pMHC II and pUMtOR-CohCR-pMHC II/DM constructed in this study.

Table S1 *Magnetospirillum magneticum* AMB-1 transformants used in this study


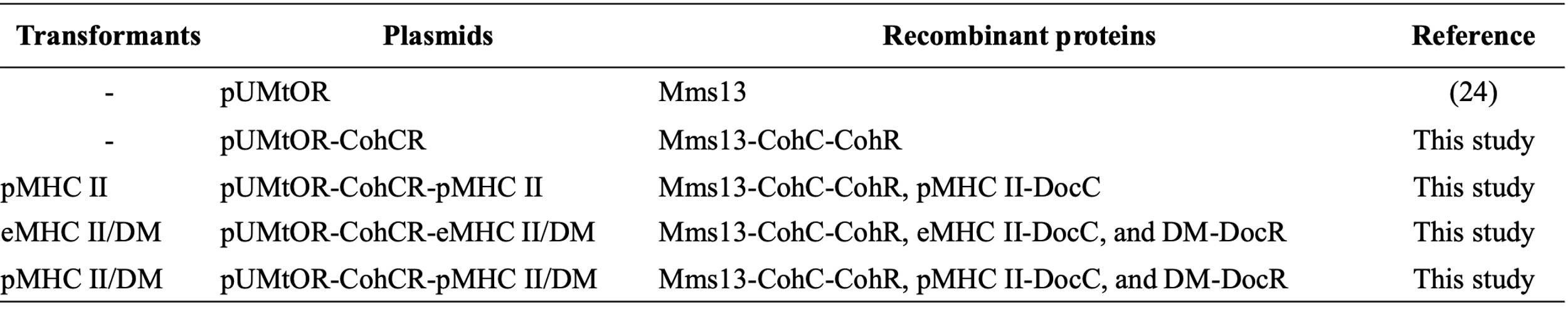


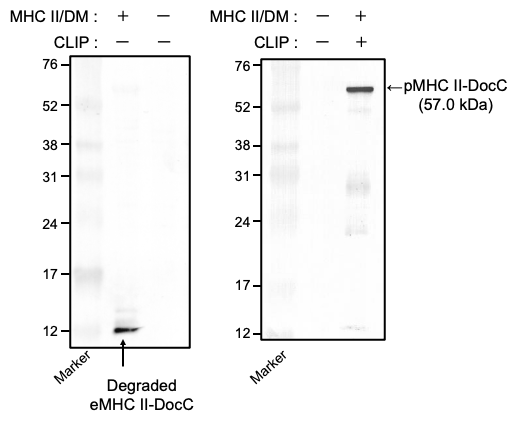


Fig. S2 Expression of MHC II-DocC on magnetosomes

Magnetosome membrane proteins from transformants expressing either empty MHC II+DM or pMHC II+DM were probed by Western blot using an anti-FLAG antibody recognizing the C-terminus of MHC II-DocC. Expected sizes are 57.0 kDa for pMHC II-DocC and 54.6 kDa for eMHC II-DocC. In the absence of the stabilizing CLIP peptide, eMHC II-DocC was largely degraded, yielding a prominent ~12 kDa fragment.
